# Predicting tuberculosis progression in school contacts: novel host biomarkers for early risk assessment

**DOI:** 10.3389/fcimb.2025.1635486

**Published:** 2025-09-01

**Authors:** Peng Lu, Meijuan Tian, Yilin Lian, Rong Wang, Xiaoyan Ding, Jingjing Pan, Hui Ding, Wei Lu, Limei Zhu, Qiao Liu

**Affiliations:** ^1^ Department of Chronic Communicable Disease, Jiangsu Provincial Center for Disease Control and Prevention, Nanjing, China; ^2^ Department of Epidemiology, Key Laboratory of Public Health Safety and Emergency Prevention and Control Technology of Higher Education Institutions in Jiangsu Province, School of Public Health, Nanjing Medical University, Nanjing, China; ^3^ Department of Epidemiology, School of Public Health, Southeast University, Nanjing, China; ^4^ Department of Tuberculosis, Nanjing Municipal Center for Disease Control and Prevention, Nanjing, China

**Keywords:** *QuantiFERON* supernatants, tuberculosis, biomarkers, progression, LASSO

## Abstract

The low positive predictive value of tuberculin skin tests and interferon-γ release assays often results in unnecessary prophylaxis. This study aimed to identify antigen-specific biomarkers with high accuracy for predicting progression to active tuberculosis (ATB). QuantiFERON supernatants from a school tuberculosis outbreak cohort were analyzed, tracking students over two years to identify ATB cases. We assessed 67 cytokines using the Luminex Multiplex Array kit and applied LASSO and multivariate logistic regression to select predictors. A nomogram was developed from the coefficients of top predictors. Model performance was evaluated by AUC, C-index, and AIC. The levels of FGFbasic, GM-CSF, MPIF-1/CCL23, as well as the combinations of ratios of FGFbasic/GM-CSF and FGFbasic/MPIF-1/CCL23 were significantly associated with the risk of ATB. AUC values for the prediction models based on individual cytokines ranged from 0.607 to 0.713, notably lower than those of the fixed models based on the logistic regression (0.932) and LASSO regression (0.939). The LASSO regression model exhibited the best predictive performance, with a higher sensitivity (0.858 vs. 0.818) and specificity (0.949 vs.0.923), lower AIC (36.323 vs. 38.232), and equivalent C-index (0.939) compared to the traditional logistic regression model. The biomarkers identified in this study offer valuable insights for developing a more precise tool to identify individuals at high risk for rapid progression to ATB disease, enabling targeted interventions. The combination of multiple immune indicators shows significant promise in improving diagnostic accuracy.

## Highlights

To our knowledge, this is the first cohort study to identify antigen-specific biomarkers with substantial predictive accuracy for adolescents at high risk of progressing to active tuberculosis. The biomarkers identified in this study pave the way for developing more precise diagnostic tools for tuberculosis, making it possible to implement targeted interventions early.

## Introduction

Tuberculosis (TB), caused by *Mycobacterium tuberculosis* (*M.tb*), likely reemerged as the world’s leading cause of death from a single infectious agent in 2023. That year, an estimated 10.8 million new TB cases were reported worldwide, with China accounting for approximately 741,000 cases, ranking third among the 30 high-burden TB countries, behind India and Indonesia. This follows a three-year period during which COVID-19 temporarily surpassed it as the most significant infectious cause of mortality (Module 1: tuberculosis surveillance, 2024). The global burden of TB remains substantial, with approximately one-fourth of the world’s population harboring a latent tuberculosis infection (LTBI) and an annual incidence of over 10.8 million new active TB (ATB) cases. Globally, TB accounts for an estimated 1.09 million deaths each year, underscoring its lasting impact on public health (World Health Organization). However, 5–10% of individuals infected with *M.tb* will progress to ATB disease within months to years after the initial infection (Organization WH, 2018). Early detection and treatment of ATB, along with preventive treatment for individuals with LTBI, are thus regarded as two fundamental pillars of TB control. These strategies are essential for achieving the TB elimination targets outlined by the World Health Organization (WHO) (Organization WH, 2014).

The diagnosis of LTBI currently lacks a definitive gold standard. The two most widely used diagnostic tools, the tuberculin skin test (TST) and interferon-γ release assays (IGRA), have limitations. Neither method can reliably differentiate LTBI from ATB, and they are associated with low positive predictive values (PPV)—approximately 2.7% for TST and 1.5% for IGRA (Diel et al., 2012). Consequently, a significant number of individuals testing positive with TST or IGRAs would need treatment to prevent progression to ATB, highlighting the need for more accurate diagnostic tools. Given the differences in treatment approaches, an immunodiagnostic test capable of distinguishing between LTBI and ATB would substantially advance clinical care. The immune response to mycobacterial infections, particularly the role of IFN-γ, has been extensively studied (Kaufmann, 2001; Ruhwald and Ravn, 2009). Urgently needed are easily measurable biomarkers or biosignatures, such as cytokines associated with the immune response to *M.tb*, to enhance the prediction of disease progression (Kaufmann, 2001; Ruhwald and Ravn, 2009; Clifford et al., 2015). These developments are crucial for enhancing global TB control efforts. Furthermore, combinations of biomarkers may offer greater sensitivity than individual markers (Nemeth et al., 2010; Frahm et al., 2011). Cytokines often synchronize to regulate inflammation, immune cell activation, and tissue repair, influencing each other’s expression through feedback mechanisms.

Despite this, existing studies mostly focus on adults, lacking dynamic immune marker analysis of LTBI progression to ATB in adolescents. We hypothesized that adolescents are optimal subjects for identifying biomarkers of TB progression due to their simpler immune profiles, which exhibit fewer comorbidities and less immune senescence than adults, thereby minimizing confounding factors in cytokine expression (Eming et al., 2017; Daniel et al., 2023). In this study, we assessed TB-antigen-stimulated cytokine levels in adolescents who advanced to ATB compared to those who did not. We aimed to reveal the distinct immune profiles between these groups and identify predictors for the transition to infectious TB disease.

## Materials and methods

### Study design

Between November 2020 and December 2021, ongoing enrollment of students in close contact with active student TB patients was conducted in Jiangsu Province. Following the “Guidelines for Tuberculosis Prevention and Control in Chinese Schools”, students were screened for *M.tb* infection and ATB, primarily using TST and Chest X-ray examinations. If the rate of students with a TST induration diameter of 10–15 millimeters exceeded the regional average, IGRAs were recommended. The residual venous blood after IGRA testing is used for cytokine detection after centrifugation, which was then stored frozen at -80 degrees Celsius. All enrolled students were followed up for two years to detect ATB. The follow-up was conducted every 6 months through symptom screening (e.g., cough, fever, weight loss) and cross-referencing with the Tuberculosis Management Information System, managed by teachers and local CDC staff. Participants were categorized into three groups: Non-LTBI, LTBI, and ATB. Subsequently, participants’ blood plasma cytokine levels were assessed through Luminex assays to tentatively identify cytokine-based biomarkers capable of distinguishing between LTBI and ATB cases. Non-LTBI students were characterized as those who were excluded from ATB and had negative IGRA results. LTBI was regarded as students who did not progress to ATB within 2 years despite having positive IGRA. ATB was diagnosed following the national guidelines.

### Multiplex chemokine assay

Chemokines in plasma were assessed utilizing the Bio-Plex Pro Human Chemokine Panel 40-plex assay kit and 27-plex assay kit. The 40-plex assay kit included 6Ckine/CCL21, BCA-1/CXCL13, CTACK/CCL27, ENA-78/CXCL5, Eotaxin/CCL11, Eotaxin-2/CCL24, Eotaxin-3/CCL26, Fractalkine/Cx3CL1, GCP-2/CXCL6, Gro-a/CXCL1, Gro-β/CXCL2, 1-309/CCL1.IP-10/CXCL10, 1-TAC/CXCL11, MCP-1/CCL2, MCP-2/CCL8, MCP-3/CCL7, MCP-4/CCL13, MDC/CCL22.MIF, MIG/CXCL9, MIP-1Q/CCL3, MIP-18/CCL15, MIP-3a/CCL20, MIP-3B/CCL19, MPIF-1/CCL23.SCYB16/CXCL16, SDF-1a+β/CXCL12, TARC/CCL17, TECK/CCL25, CXCL8, IFN-y, IL-1β, IL-2, 1L-4, 1L-6, IL-10, IL-16, GM-CSF and TNF-α. The 27-plex assay kit included FGFbasic, Eotaxin, G-CSF, GM-CSF, IFN-y, IL-1B, IL-1ra, 1L-2, 1L-4, 1L-5, 1L-6, 1L-7, 1L-8, 1L-9, 1L-10, IL-12(p70), 1L-13, 1L-15, 1L-17, IP-10, MCP-1(MCAF), MIP-1Q, MIP-1B, PDGF-BB, RANTES, TNF-α and VEGF. Samples, standards, and blank controls were incubated on a shaker at 850 rpm for 30 minutes. Each detection batch included internal controls and duplicate samples to ensure reproducibility and consistency.

The diluted microspheres are first distributed into a 96-well plate, followed by the addition of standards, samples, and blank controls. The plate is then incubated on a plate shaker at 850 rpm for 30 minutes at room temperature. After removing the samples, the plate is washed three times using a plate washer. The detection antibody is diluted with Antibody Diluent according to the instructions, and 25 µL of the diluted antibody is added to each well. The plate is covered and incubated on the shaker at 850 rpm for 30 minutes at room temperature, in the dark. Following the detection antibody incubation, the plate is washed three times, and Streptavidin-PE is diluted with Assay Buffer as per the instructions. 50 µL of the diluted Streptavidin-PE is added to each well, and the plate is incubated on the shaker at 850 rpm for 10 minutes at room temperature, in the dark. After three additional washes, 125 µL of Assay Buffer is added to each well to resuspend the microspheres. The plate is then incubated for 2 minutes on the shaker at 850 rpm, in the dark, before being read using a calibrated Luminex machine.

### Diagnostic tests for LTBI

TST was performed using the Mantoux method (Lu et al., 2023). QuantiFERON-TB Gold-in Tube (QFT) Assay was used to evaluate LTBI. Whole blood samples were collected and incubated in three different tubes: one with TB antigen (stimulated), one with mitogen (positive control), and one without any antigen (unstimulated, Nil). The processing followed the manufacturer’s guidelines (QIAGEN, Germany). After incubation, the supernatants were collected to measure the interferon-γ (IFN-γ) response, expressed in IU/mL. The results were analyzed using the QFT software to determine whether the individuals were QFT-positive or negative. Any remaining supernatants were promptly stored at −80 °C for future analysis.

### Detection of ATB

Two complementary approaches were employed to identify ATB among study participants. The first involved active follow-up conducted by teachers and local staff from the Center for Disease Control and Prevention. The second was a retrospective case detection based on registry data. ATB cases were identified by cross-referencing two datasets: (1) records containing information on all study participants and (2) the Tuberculosis Management Information System of Jiangsu Province, which documented all reported TB cases from 2020 to 2022. To link individuals between the cohort and the TB registry database, we utilized identifiers such as name, date of birth, sex, address, and identification number.

### Statistical analysis

Standard 2 × 2 contingency tables, the interquartile range (IQR), and mean ± standard deviation (SD) were employed for summarizing categorical and continuous variables, respectively. Fisher’s exact test was utilized to compare the frequencies of categorical variables. For quantitative data, statistical analysis was performed using non-parametric tests (Mann-Whitney U for two groups, Kruskal-Wallis for multiple groups). Differences in chemokine levels between group pairs were compared using one-way ANOVA. Subsequently, we compared two models: a conventional Logistic Regression model and a model based on the Least Absolute Shrinkage and Selection Operator (LASSO) regression. In the logistic regression model, we applied forward selection, a variable selection method where predictors are iteratively added based on their statistical significance (P < 0.10). LASSO regression analysis, employing 10-fold cross-validation, was utilized to reduce dimensionality and identify the most significant predictors. The λ value in LASSO regression was selected using the one standard error (1SE) rule to balance model simplicity and predictive accuracy. Due to the limited sample size, no separate training and validation sets were created; instead, 10-fold cross-validation was used to evaluate model performance. A nomogram was then developed by integrating all the most valuable predictors (MVPs) based on their regression coefficients from the binary logistic regression (Friedman et al., 2010). The nomogram’s discrimination capability was evaluated using the area under the receiver operating characteristic curve (AUC), the concordance index (C-index), and the Akaike Information Criterion (AIC) (Hanley and McNeil, 1982).

Additionally, decision curve analysis (DCA) was conducted to evaluate the net clinical benefit of the nomogram (Vickers and Elkin, 2006). DCA assesses the net clinical benefit of the predictive model across different threshold probabilities to guide clinical decision-making. The ability of potential predictive biomarkers to differentiate between groups was assessed using principal component analysis (PCA). A *P*-value less than 0.05 was considered statistically significant. Data were analyzed using R software version 4.3.2 (https://www.r-project.org).

### Ethics approval and consent to participate

This study was reviewed and approved by the ethics committee of the Center for Disease Control and Prevention of Jiangsu Province. All eligible participants signed written informed consent (Approval Number: JSJK2024-B022-01).

## Results

### Study population

This study initially enrolled a total of 62 students, with 12 individuals excluded from the initial cohort due to insufficient supernatant availability for analysis, resulting in a final sample size of 50 participants. Of the 50 participants, 21 individuals (42%) were tested negative on the QFT assay, while 29 participants (60%) yielded positive results. After the following 2 years, 11 participants developed ATB (**Supplementary Table 1**), with 10 exhibiting positive QFT results. The median age of the entire cohort was 21 years (IQR: 20-24). Among the participants, 36 individuals (72.0%) were female. TST reaction diameter for all 50 individuals was 13.0 mm (IQR: 4.5-15.0) (**Table 1** and **Supplementary Table 2**).

**Table 1:** Demographic characteristics of the 50 participants, overall and by QuantiFERON-TB Gold In-Tube test status.

Variable	All	QFT				
		Negative	Positive without tuberculosis	Positive with tuberculosis	Statistic	*P*
Sex						
Female	36 (72)	15 (75)	15 (79)	6 (55)	Fisher-exact	0.428
Male	14 (28)	5 (25)	4 (21)	5 (45)		
Age (years)	21 (20, 24)	26 (20, 35)	21 (20, 21)	20 (20, 22)	Kruskal-W, 7.7	0.021
TST (mm)	13.0 (4.5, 15.0)	10.25 (0.0, 14.75)	14.00 (13.00, 15.50)	12.50 (0.00, 15.50)	Kruskal-W, 4.5	0.105
TB Ag-Nil	0.68 (0.01, 2.15)	0.01 (-0.01, 0.07)	1.85 (0.79, 3.63)	1.59 (0.24, 3.01)	Kruskal-W, 30.1	<0.001
FGFBasic	11.47 (9.39, 13.43)	11.47 (9.91, 13.43)	10.44 (9.39, 11.47)	15.32 (11.47, 17.13)	Kruskal-W, 6.1	0.048
Eotaxin	85.48 (66.64, 135.94)	89.11 (72.98, 144.48)	68.58 (55.91, 92.11)	97.68 (73.19, 143.46)	Kruskal-W, 4.9	0.086
GM-CSF	1.53 (1.13, 1.83)	1.42 (0.67, 1.56)	1.55 (1.53, 2.34)	1.42 (1.13, 1.56)	Kruskal-W, 5.4	0.066
IL-10	0.67 (0.32, 0.95)	0.45 (0.18, 0.71)	0.95 (0.45, 1.25)	0.70 (0.32, 0.83)	Kruskal-W, 5.0	0.083
IL-16	116.95 (85.69, 164.66)	96.58 (67.46, 120.20)	117.69 (101.39, 167.18)	139.24 (116.95, 222.16)	Kruskal-W, 6.6	0.036
I-309/CCL1	24.36 (19.55, 30.83)	24.36 (22.62, 25.38)	24.03 (8.89, 27.91)	24.36 (17.79, 33.56)	Kruskal-W, 1.7	0.431
MPIF-1/CCL23	74.90 (52.42, 74.90)	75.45 (65.81, 87.87)	75.45 (52.89, 92.40)	52.73 (32.23, 66.41)	Kruskal-W, 7.1	0.029

### Comparisons of chemokines

**Figure 1:**
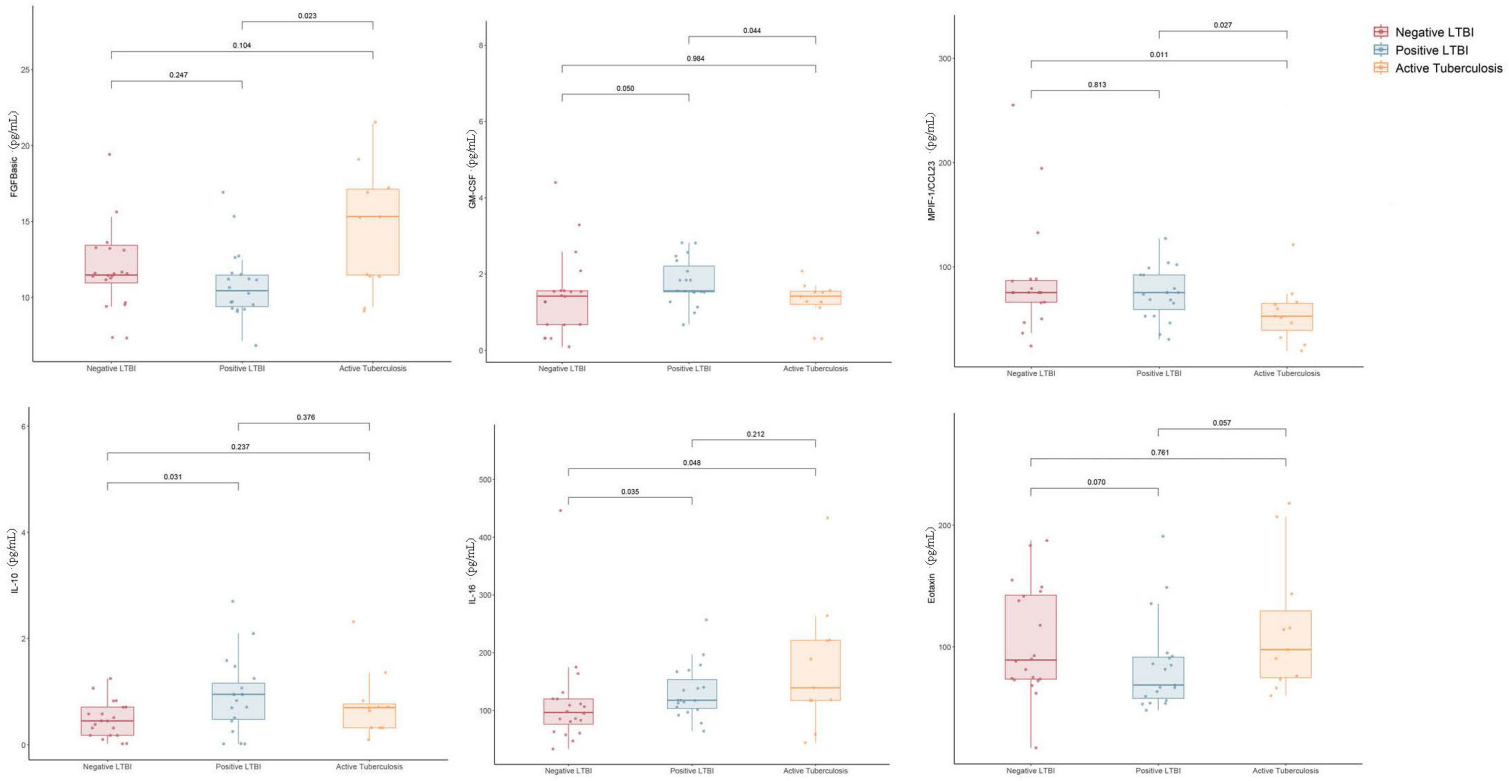
Difference in plasma cytokines levels between negative LTBI, positive LTBI and ATB by Luminex.

Grouped according to QFT results, we found that the baeline levels of IL-10 (Z = -2.295, P = 0.022) and IL-16 (Z = -1.976, P = 0.048) were significantly higher in the positive QFT group compared to the negative QFT group. However, when individuals were grouped into the negative QFT group, positive QFT group, and ATB group, in addition to IL-10 (W = 113.5, P = 0.032) and IL-16 (W = 115, P = 0.036) was also significantly higher in the positive QFT group compared to the negative QFT group (**Figure 1**).

**Figure 2:**
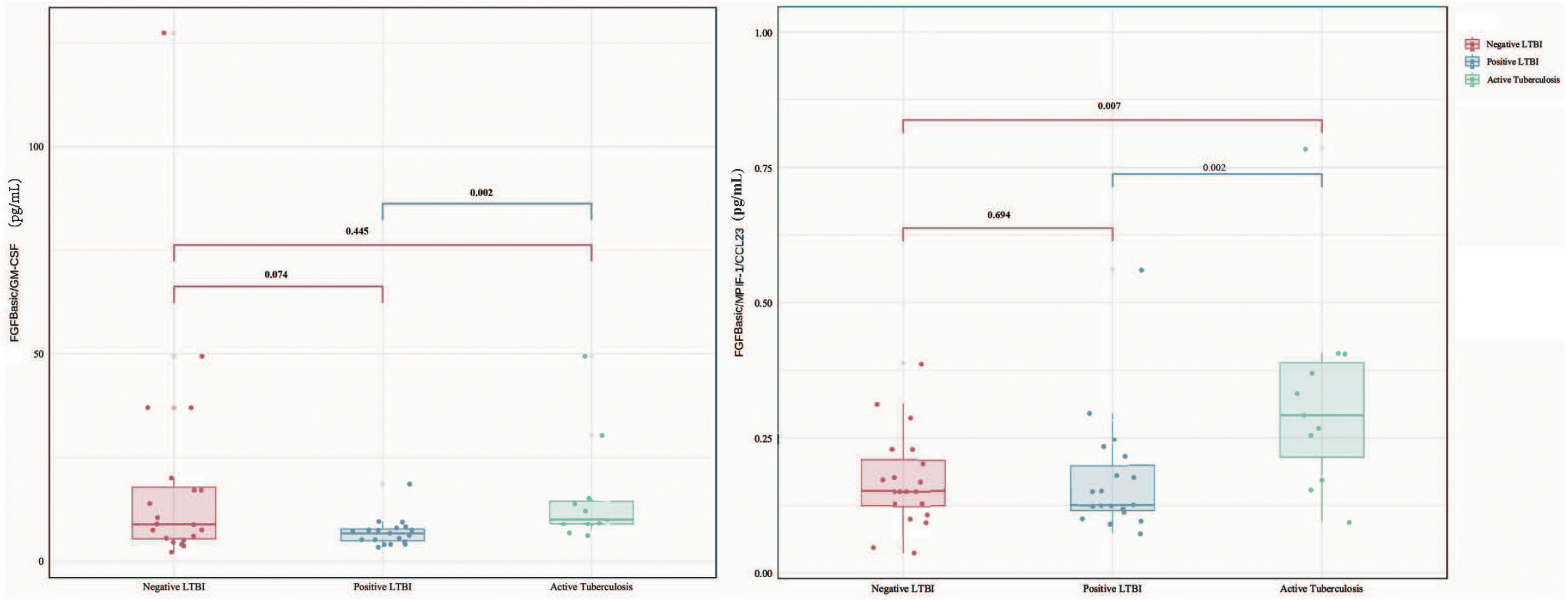
Difference in plasma cytokines levels between negative LTBI, positive LTBI and ATB by Luminex among FGFbasic/GM-CSF and FGFbasic/MPIF-1/CCL23.

When comparing cytokine levels between the ATB group and the LTBI group, the ATB group exhibited higher levels of FGFbasic (W = 52.5, P = 0.023) but lower levels of GM-CSF (W = 151, P = 0.046) and MPIF-1/CCL23 (W = 156, P = 0.028) (**Figure 1**). Thus, the ratios of FGFbasic/GM-CSF (W = 223, P = 0.002) and FGFbasic/MPIF-1/CCL23 (W = 231, P = 0.006) emerged as highly promising risk signatures for predicting the risk of progression from LTBI to ATB (**Figure 2**).

**Figure 3:**
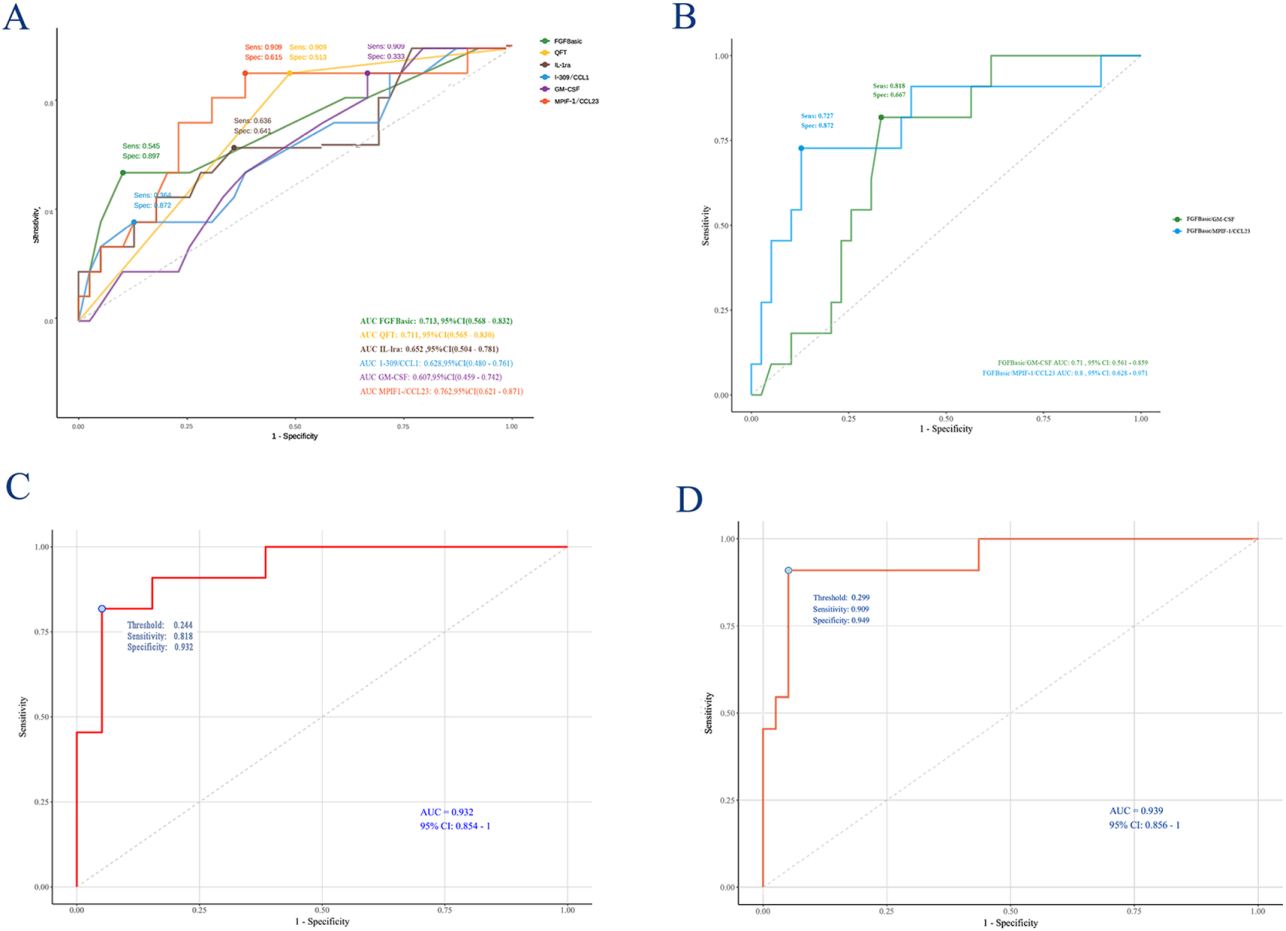
Receiving operating characteristic curve analysis between TB and non-TB. AUC, area under the curve. **(A)** FGFbasic, GM-CSF, FGFbasic, IL-1ra, I-309/CCL1, MPIF-1/CCL23, FGFbasic/I-309/CCL ratio, FGFbasic/MPIF-1/CCL23; **(B)** FGFbasic/GM-CSF and FGFbasic/MPIF-1/CCL23; **(C)** Logistic Regression model; **(D)** Lasso model.

The AUC values (95% CI) for Baseline FGF, QFT, IL-1ra, I-309/CCL1, GM-CSF, and MPIF-1/CCL23 were 0.713 (0.568–0.832), 0.711 (0.565–0.830), 0.652 (0.504–0.781), 0.628 (0.480–0.761), 0.607 (0.459–0.742), and 0.762 (0.621–0.871), respectively (**Figure 3A**). Additionally, the AUC values for the combinations of FGFBasic/GM-CSF and FGFBasic/MPIF-1/CCL23 were 0.71 (0.561–0.859) and 0.80 (0.628–0.971), respectively (**Figure 3B**, **Supplementary Table 3**).

### Identification of optimal predictive factors for ATB through logistic regression model

A final Logistic Regression analysis was conducted using a model selected through Stepwise Forward Selection, which included four predictors: QFT, FGFbasic, IL-1ra, and I-309/CCL1. The fitted coefficients and OR for each predictor in the model are as follows: QFT (OR = 16.08, 95% CI: 1.83, 447.05, P=0.034), FGFbasic (OR = 1.58, 95% CI: 1.19, 2.37, P=0.006), IL-1ra (OR = 1.01, 95% CI: 1.00, 1.02, P=0.137), and I-309/CCL1 (OR = 10.92, 95% CI: 0.83, 1.00, P=0.076) (**Table 2**). In this Logistic Regression model, the AUC for predicting ATB was 0.932 (95% CI: 0.854, 1.000), with a C-index of 0.939 and an AIC of 38.232. The model demonstrated a sensitivity of 0.818 and a specificity of 0.923 (**Figure 3C**; **Supplementary Tables 3, 4**). A correlation heat map is presented in **Supplementary Figure 1**, where we identified several chemokines with strong correlations.

**Table 2:** Coefficients, Odd Ratios, and 95% Confidence Intervals of the predictors in the Final two Models.

Factors	Logistic regression model			Lasso regression model		
	Coefficients	OR (95% CI)	*P*	Coefficients	OR (95% CI)	*P*
QFT						
Negative	Reference	Reference	00.034	Reference	Reference	0.135
Positive	2.778	16.08 (1.83, 447.05)		1.974	7.20 (0.73, 196.61)	
BasicFGF	0.46	1.58 (1.19, 2.37)	0.006	0.402	1.50 (1.11, 2.30)	0.021
IL-1ra	0.007	1.01 (1.00, 1.02)	0.137	0.008	1.01 (1.00, 1.02)	0.063
I-309/CCL1	-0.081	0.92 (0.83, 1.00)	0.076	-0.065	0.94 (0.84, 1.01)	0.14
MPIF-1/CCL23	–	–	–	-0.049	0.95 (0.89, 1.00)	0.081

### Identification of optimal predictive factors for ATB through the LASSO regression model

**Figure 4:**
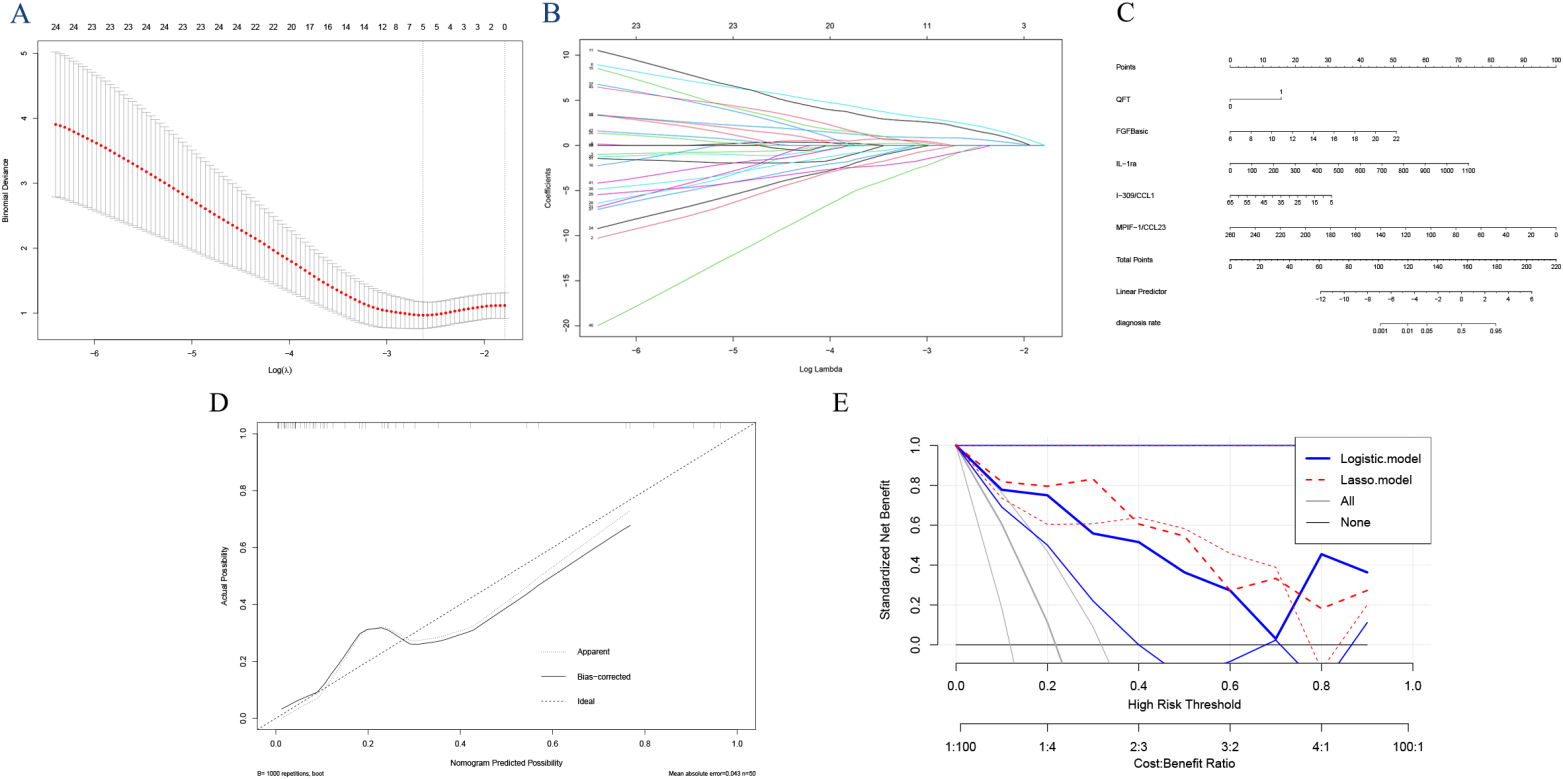
Feature selection using the least absolute shrinkage and selection operator (LASSO) analysis with 10-fold cross-validation. Lambda (tuning parameter) selection of deviance in the LASSO regression based on the one standard error criteria (right dotted line) and the minimum criteria (left dotted line) **(A)**. LASSO coefficient profiles of the candidate features. The intersecting curves represent the number of features retained at that log (lambda) value. **(B)** “FGFbasic,” “GM-CSF,” “IL-1ra,” “I-309/CCL1,” and “MPIF-1/CCL23”. **(C)** Nomogram for predicting the risk of tuberculosis outcomes. **(D)** the calibration curve. **(E)** and the decision curve analysis.

Applying LASSO regression with 10-fold cross-validation, we determined the optimal hyperparameter λ based on the bivariate deviation. Utilizing this optimal λ, five nonzero coefficients of preoperative features were identified as the MVPs for ATB (**Figures 4A, B**). The identified factors, including QFT, FGFbasic, IL-1ra, I-309/CCL1, and MPIF-1/CCL23, were recognized as potential prognostic indicators for the development of ATB at the optimal λ value of 0.083. These variables were then incorporated into the multivariate logistic regression analyses. The findings indicated that positive QFT results (OR=7.20, 95% CI: 0.73, 196.61, P=0.135), elevated levels of FGFbasic (OR=1.50, 95% CI: 1.11, 2.30, P=0.021), and IL-1ra (OR=1.01, 95% CI: 1.00, 1.02, P=0.063), along with decreased levels of I-309/CCL1 (OR=0.94, 95% CI: 0.84, 1.01, P=0.140) and MPIF-1/CCL23 (OR=0.95, 95% CI: 0.89, 1.00, P=0.081) were independent prognostic factors for the progression to ATB (**Table 2**).

### Establishment of the nomogram

Using the coefficients of the five predictors (1.974, 0.402, 0.008, -0.065, and -0.049), we created a nomogram with the ‘rms’ package in R software (**Figure 4C**). The lengths of the lines in the nomogram represent the importance of each predictor. Among these, MPIF-1/CCL23 had the highest weight, indicating it is the most significant predictor of TB, followed by FGFbasic, IL-1ra, and I-309/CCL1. Conversely, QFT was identified as the least influential factor. The total score was calculated by summing the preoperative scores for the five relevant symptoms. The risk of developing ATB was assessed by drawing a vertical line from the total score to the risk scale. The AUC (0.9394, 95% CI: 0.857-1.000) and C-index (0.939) indicated good calibration and accuracy, respectively (**Figure 3D**). The calibration and DCA curves also displayed close alignment between the observed and predicted probabilities (**Figure 4D, E**; **Supplementary Tables 3, 4**).

We performed principal component analysis (PCA) on the five analytes (“FGFbasic,” “GM-CSF,” “IL-1ra,” “I-309/CCL1,” and “MPIF-1/CCL23”) that were identified as significantly different through dominant analysis, to assess their ability to differentiate between ATB and non-ATB (**Supplementary Figure 2**). The PCA results demonstrated a clear and distinct separation of the two groups, highlighting the effectiveness of these biomarkers in distinguishing between them.

### Comparison of the different models

The AUCs of the prediction models based on individual cytokines ranged from 0.607 to 0.713, notably lower than those of the fixed models utilizing Logistic regression (AUC: 0.932) and Lasso regression (AUC: 0.939). Among these, the Lasso regression model demonstrated superior predictive performance, with higher sensitivity (0.858 vs. 0.818), identical specificity (0.949), lower AIC (36.323 vs. 38.232), an equivalent C-index (0.939), and better performance in the DCA (**Figure 4E**) compared to the traditional logistic regression model. The 10-fold cross-validated AUC of the LASSO model was 0.960 (95% CI: 0.901–1.000), while that of the logistic regression model was 0.962 (95% CI: 0.908–1.000), demonstrating strong and stable discriminative ability for both models. These results indicate that the Lasso regression model outperformed the other two models in predicting TB. Meanwhile, the hazard ratio(HR) for progression to ATB according to the LASSO model was 66.85, nearly twice as high as the 38.98 calculated using the Logistic model (**Supplementary Table 5**).

## Discussion

While IGRAs generally exhibit limited prognostic capability, the IGRA test can effectively identify additional biomarkers. Stimulating T cells with *M.tb* antigens triggers a surge in a complex network of cytokines, reflecting the immune responses specific to TB. In this study, we conducted an exploratory analysis of soluble biomarkers to predict progression to ATB. We utilized single and combined cytokine biomarkers through LASSO regression and multivariate logistic regression analysis to identify significant factors distinguishing ATB from non-TB cases. Our findings revealed that the predictive ability of single cytokines was relatively low. However, this predictive capability significantly improved when combined with positive QuantiFERON test results and was greatly enhanced using combined cytokine biomarkers through LASSO regression and multivariate Logistic regression analysis.

IGRAs have shown reliable performance in detecting LTBI, making them valuable adjuncts in diagnosing ATB, with superior accuracy over the TST in predicting ATB. However, their limited ability to distinguish ATB from non-TB cases reduces their clinical utility (Auguste et al., 2017; Lu et al., 2021; Hamada et al., 2023). Host biomarker studies have been at the center of interest for TB immunodiagnostics due to their utility in developing a point-of-care assay. In this study, we undertook an exploratory analysis of soluble biomarkers capable of predicting the progression of TB disease. To contextualize our findings, we compared them with studies employing diverse methodologies to identify predictive biomarkers for TB progression (Lacombe et al., 2014; Penn-Nicholson et al., 2019; Daniel et al., 2023). Our results align with and extend previous research using QuantiFERON-based, transcriptomic, proteomic, and multi-omic approaches. For example, Daniel et al. (2023) identified IP-10, CCL19, IFN-γ, IL-1ra, CCL3, and GM-CSF in QuantiFERON supernatants as predictors of ATB in household contacts, consistent with our identification of FGFbasic and MPIF-1/CCL23. Similarly, Penn-Nicholson et al. (2019)) reported a proteomic signature(TRM5 and 3PR)for TB progression, supporting the role of inflammatory markers akin to our cytokine ratios. Transcriptomic analyses by Gupta et al. (Ji et al., 2019) and Roe et al. (Sanchez et al., 2023) identified gene expression profiles linked to immune activation, which align with our cytokine networks. Multi-omic studies by Huang et al. (Scriba and Mendelsohn, 2020) integrate proteomic and transcriptomic data to enhance predictive accuracy, reinforcing the superiority of our combined biomarker models. Rodríguez-Molino et al. (2024)) reported higher rates of nonrespiratory TB and severe disease in immunocompromised children, highlighting the poor sensitivity of immune-based tests like QFT, which supports our focus on novel biomarkers to improve diagnostic accuracy in high-risk groups. These studies collectively suggest that our LASSO-derived biomarkers could be tested in broader populations, including immunocompromised individuals, to confirm their predictive utility. These datasets provide opportunities for future validation of our nomogram across diverse cohorts, potentially broadening its clinical applicability. We found a significant difference in the levels of 3 analytes (FGFbasic, GM-CSF, and MPIF-1/CCL23) of the 67 and 2 combinations (FGFbasic/GM-CSF and FGFbasic/MPIF-1/CCL23) in progressors as compared to non-progressors. Macrophages produce FGF-basic and plays a role in mitogenic and angiogenic activity (Henke et al., 1993). While its association with TB has not been consistently established (Awoniyi et al., 2016; Sariko et al., 2019), increased expression of FGFbasic may contribute to mechanisms that promote TB pathogenesis, although the exact mechanisms remain unclear. FGFbasic has been implicated in other inflammatory diseases, such as rheumatoid arthritis and pulmonary fibrosis, where it modulates inflammation and tissue remodeling, suggesting a potential role in TB progression through pathways like granuloma formation and fibrosis (Gudbjörnsson et al., 2004; Ghanem et al., 2024).

During LTBI, FGFbasic could potentially support the establishment of immune tolerance, inhibiting the immune system’s ability to clear *M.tb*. By modulating cytokine balance, FGFbasic may help the immune system maintain “tolerance” to the pathogen, allowing *M.tb* to persist in the host. This could facilitate the transition from LTBI to ATB. Indirect support for this hypothesis is provided by a study showing a decrease in FGFbasic expression following the initiation of TB treatment. GM-CSF is a growth factor that acts as a chemoattractant, recruiting neutrophils, lymphocytes, and macrophages. It plays a crucial role in supporting the inflammatory response during the productive phases (Olsson et al., 2006; Cheknev et al., 2014) and is involved in granuloma formation in TB to contain the infection (Szeliga et al., 2008). Previous studies show that lower GM-CSF expression may serve as a potential biomarker for predicting TB, although the underlying mechanism remains unclear (Balcells et al., 2018; Sariko et al., 2019).

In contrast, reduced levels of MPIF-1/CCL23 in progressors suggest a disruption in granuloma formation, which facilitates disease progression—this finding diverges from previous studies (Li et al., 2023; Yang et al., 2023). Pang et al. and Zhang et al. observed higher expression of MPIF-1/CCL23 in ATB cases compared to non-TB controls, though without statistical significance, proposing it as a predictive biomarker for TB (Li et al., 2023; Yang et al., 2023). This discrepancy may be explained by the possibility that the immune profile of the ATB group is in an early or “standby” state, which could contribute to TB development (Yang et al., 2023). The distinct immune profiles observed at different disease stages suggest that MPIF-1/CCL23 may have a stronger predictive ability for the progression to ATB. Our study identified the FGFbasic/GM-CSF and FGFbasic/MPIF-1/CCL23 ratios as highly promising predictive markers for ATB, outperforming single markers. However, further studies are needed to validate these marker combinations in their respective cohorts.

The study population included a higher proportion of female participants, which may influence immune marker expression. Female hormones, such as estrogen, have been shown to modulate immune responses, potentially affecting cytokine production and TB progression. For instance, estrogen can enhance macrophage activation and cytokine release, which may explain the observed differences in FGFbasic and GM-CSF levels in our cohort (Straub, 2007; Kovats, 2015). Further studies are needed to explore gender-specific immune profiles in TB progression.

The host response to LTBI and ATB involves a complex network of cytokines and chemokines at various levels of the immune system. Other diseases can also trigger a broad array of cytokines and chemokines, complicating the interpretation of infection status. Several studies have suggested using a multiplex model of cytokines and chemokines as biomarkers to differentiate TB infection states, offering improved sensitivity and specificity compared to single cytokine or chemokine tests (Wang et al., 2012; Yang et al., 2023). Through logistic regression, we identified a combination of “QFT, FGFbasic, IL-1ra, and I-309/CCL1” and increased the AUC to 0.932, sensitivity to 0.818, and specificity to 0.923. Compared to traditional logistic regression, LASSO regression offers key advantages such as automatic variable selection, reduced overfitting, better handling of multicollinearity, and model simplification for more straightforward interpretation. Additionally, LASSO improves predictive performance, especially in high-dimensional data (Sukarti et al., 2024). Our study also confirmed that, compared to logistic regression, the sensitivity for predicting ATB was increased to over 0.9 as it achieves the optimal requirement of ≥90% sensitivity for biomarker-based non-sputum tests to predict TB progression per the target product profile (Penn-Nicholson et al., 2019; Daniel et al., 2023).

An intriguing observation is that the multivariate models include variables that do not show statistical significance when considered individually, such as QFT, MPIF-1/CCL23, IL-1ra, and I-309/CCL1. Like IGRAs, which have limited prognostic ability and a low positive predictive value, the IGRA test may still be valuable for identifying additional biomarkers to improve predictive accuracy (Daniel et al., 2023). The decision tree analysis further supported that cytokine predictive capabilities are enhanced in individuals with positive QFT results. Additionally, IL-1ra, an antagonist of IL-1 produced during inflammatory processes, has been shown in previous studies to be associated with increased susceptibility to LTBI and elevated levels in ATB cases (Ji et al., 2019; Sanchez et al., 2023). Notably, IL-1ra was detectable in the serum of LTBI patients and found to be at lower levels in those treated for LTBI compared to untreated individuals, suggesting its potential as a marker for the progression to active disease (Juffermans et al., 1998; Zhang et al., 2020).

Our study has several notable strengths. First, the cohort consisted entirely of adolescents, which helped avoid potential cross-reaction with cytokines from other chronic diseases. Second, all participants were from a school outbreak, indicating that LTBI was likely recent. Third, the nomogram was tested using independent cohorts from different centers, enhancing its reliability and generalizability to a large extent.

However, we acknowledge several limitations in the current study. First, the small sample size (n=50, with 11 active tuberculosis [ATB] cases) limits statistical power, reducing the precision and generalizability of our findings. This restricted cohort may lead to overfitting in the LASSO model, despite 10-fold cross-validation, and could bias biomarker selection, potentially compromising the nomogram’s robustness. The limited number of outcome events further restricts our ability to detect subtle differences in cytokine profiles. Larger, multi-center studies with diverse populations are essential to validate our findings, enhance statistical power, and ensure robust biomarker performance. Second, the LASSO logistic regression model has not been independently validated in other cohorts. Finally, we cannot rule out the possibility that some individuals in the ATB group may have been in the subclinical or early stages of TB. Third, event times for ATB development were not recorded, limiting our ability to perform time-to-event analyses such as Cox regression. This represents a methodological limitation of using logistic regression, which does not account for the timing of disease progression.

To address the need for multi-center cohort validation, we plan to collaborate with regional TB control centers in Jiangsu and neighboring provinces to recruit a larger, diverse cohort for external validation. This will involve prospective enrollment of adolescents and adults with recent LTBI exposure, using standardized IGRA and cytokine assays, and longitudinal follow-up for at least 2 years to confirm the predictive accuracy of our nomogram.

## Conclusions

Our study identified soluble TB-specific biomarkers that could be effectively used as potential short-term risk predictors for TB. Specifically, ratios such as FGFbasic/GM-CSF and FGFbasic/MPIF-1/CCL23 may serve as promising markers for predicting the progression from non-TB to ATB, particularly in individuals with positive IGRA results. Additionally, the combination of multiple immune indicators analyzed through LASSO logistic regression demonstrated significant potential in enhancing the diagnosis of ATB.

## Data Availability

The data analyzed in this study is subject to the following licenses/restrictions: The datasets used and/or analysed during the current study are available from the corresponding author on reasonable request. Requests to access these datasets should be directed to Peng Lu, lpjscdc@163.com.

## Supplementary material

Supplementary Figure 1 Correlation Heatmap for potential predictive biomarkers. **(A)** Correlation Heatmap; **(B)** Significant Correlations (P < 0.05).

Supplementary Figure 2 CPCA plot of significantly different biomarkers between tuberculosis and non-progressors: PCA shows that FGFbasic, GM-CSF, FGFbasic, IL-1ra, I-309/CCL1, MPIF-1/CCL23 can clearly distinguish between tuberculosis and non-tuberculosis with no overlap.
