# Supplementary material for: Predicting tuberculosis progression in school contacts: novel host biomarkers for early risk assessment
Source: Front Cell Infect Microbiol. 2025 Sep 1;15:1635486. doi: 10.3389/fcimb.2025.1635486 (PMC12434022; doi:10.3389/fcimb.2025.1635486)
Supplement: Supplementary Figure 1 — Correlation Heatmap for potential predictive biomarkers. (A) Correlation Heatmap; (B) Significant Correlations (P < 0.05). [file DataSheet1.pdf]

Supplement Table 1. The Expression of Biomarkers in 11 Tuberculosis Patients

Tuberculosis	TST	QFT	BasicFGF	IL-1ra	GM-CSF	I-309/CCL1	BasicFGF/ GM-CSF	BasicFGF/ MPIF-1/CCL23	predicted_LASSO model	Predicted_logistic model
Index 1	1	1	9.39	152.27	1.53	5.04	6.14	0.37	0.56	0.25
Index 2	0	1	9.39	382.17	0.31	27.91	30.29	0.29	0.58	0.21
Index 3	1	1	11.47	169.31	1.69	34.59	6.79	0.15	0.04	0.08
Index 4	1	1	11.47	235.03	1.28	11.67	8.96	0.17	0.34	0.48
Index 5	0	1	11.47	1014.94	1.28	5.04	8.96	0.09	0.97	1.00
Index 6	1	1	15.32	215.92	1.53	24.36	10.01	0.79	0.90	0.63
Index 7	1	1	15.32	708.08	0.31	24.36	49.42	0.26	0.99	0.98
Index 8	0	0	17.13	271.07	1.42	22.62	12.06	0.27	0.34	0.29
Index 9	1	1	17.13	312.99	1.13	8.89	15.16	0.33	0.96	0.96
Index 10	1	1	18.89	140.34	2.08	24.03	9.08	0.41	0.84	0.84
Index 11	1	1	21.43	163.73	1.56	30.27	13.74	0.41	0.90	0.92

Supplement Table 2. Demographic characteristics of the 50 participants, overall and by Tuberculosis and Non-tuberculosis

Variables	Total (n = 50)	Non-tuberculosis (n = 39)	Tuberculosis (n = 11)	P Value
sex, n (%)				0.252
Female	36 (72)	30 (77)	6 (55)	
Male	14 (28)	9 (23)	5 (45)	
age, Median (Q1,Q3)	0.11 (0.07, 0.14)	0.11 (0.07, 0.45)	0.07 (0.07, 0.11)	0.034
TST, Median (Q1,Q3)	0.63 (0.32, 0.73)	0.66 (0.35, 0.73)	0.61 (0.27, 0.74)	0.85
TB Ag-Nil, Median (Q1,Q3)	0.11 (0.03, 0.28)	0.06 (0.03, 0.24)	0.22 (0.11, 0.36)	0.122
QFT, n (%)				0.016
Negative	21 (42)	20 (51)	1 (9)	
Positive	29 (58)	19 (49)	10 (91)	
BasicFGF, Median (Q1,Q3)	0.3 (0.16, 0.44)	0.3 (0.16, 0.34)	0.57 (0.3, 0.7)	0.028
Eotaxin, Median (Q1,Q3)	0.34 (0.25, 0.57)	0.32 (0.25, 0.54)	0.4 (0.29, 0.56)	0.227
IFN-γ, Median (Q1,Q3)	0.15 (0.1, 0.23)	0.15 (0.1, 0.2)	0.28 (0.11, 0.33)	0.216
IL-1beta, Median (Q1,Q3)	0.87 (0.87, 1.22)	0.87 (0.87, 1.22)	1.22 (0.87, 1.22)	0.083
IL-1ra, Median (Q1,Q3)	195.91 (152.27, 264.49)	195.91 (140.34, 244.29)	235.03 (163.73, 382.17)	0.127
IL-12, Median (Q1,Q3)	11.31 (11.31, 18.95)	11.31 (11.31, 18.95)	11.31 (11.31, 18.95)	0.933
IL-13, Median (Q1,Q3)	0.61 (0.09, 0.61)	0.61 (0.09, 0.61)	0.61 (0.09, 1.06)	0.247
IL-17, Median (Q1,Q3)	3.82 (2.24, 3.82)	3.82 (2.24, 3.82)	3.82 (2.24, 5.37)	0.581
IL-4, Median (Q1,Q3)	0.47 (0.39, 0.63)	0.47 (0.37, 0.63)	0.51 (0.39, 0.65)	0.339
IL-8, Median (Q1,Q3)	0.06 (0.02, 0.13)	0.07 (0.03, 0.13)	0.04 (0.01, 0.16)	0.648
IL-9, Mean±SD	0.43±0.22	0.42±0.24	0.45±0.16	0.672
IP-10, Median (Q1,Q3)	0.33 (0.17, 0.52)	0.35 (0.17, 0.54)	0.31 (0.19, 0.52)	0.806
MCP-1, Median (Q1,Q3)	0.24 (0.17, 0.36)	0.27 (0.17, 0.36)	0.21 (0.18, 0.31)	0.59
MIP-1alpha, Median (Q1,Q3)	0.13 (0.06, 0.21)	0.1 (0.06, 0.19)	0.15 (0.08, 0.32)	0.508
MIP-1beta, Mean±SD	0.39±0.23	0.38±0.22	0.44±0.26	0.5
PDGF-BB, Median (Q1,Q3)	542.48 (302.36, 740.20)	467.49 (302.36, 686.69)	667.33 (561.06, 1012.82)	0.114
RANTES, Mean±SD	0.3±0.2	0.31±0.22	0.26±0.11	0.3
TNF-alpha, Median (Q1,Q3)	93.75 (78.82, 118.22)	88.80 (78.82, 108.49)	98.69 (78.82, 118.22)	0.298
GCP-2/CXCL6, Mean±SD	0.39±0.21	0.37±0.21	0.44±0.22	0.384
GM-CSF, Median (Q1,Q3)	1.53 (1.13, 1.83)	1.53 (1.13, 2.08)	1.42 (1.13, 1.56)	0.279
Gro-beta/CXCL2, Median (Q1,Q3)	0.24 (0.16, 0.37)	0.24 (0.16, 0.37)	0.24 (0.24, 0.25)	0.595
I-309/CCL1, Median (Q1,Q3)	24.36 (19.55, 30.83)	24.36 (19.55, 32.49)	24.03 (8.89, 27.91)	0.195
I-TAC/CXCL11, Mean±SD	0.4±0.22	0.4±0.22	0.37±0.25	0.753
IL-10, Median (Q1,Q3)	0.67 (0.32, 0.95)	0.58 (0.25, 0.95)	0.70 (0.32, 0.83)	0.851
IL-16, Median (Q1,Q3)	0.2 (0.13, 0.3)	0.19 (0.13, 0.25)	0.26 (0.2, 0.46)	0.073
IL-1beta, Mean±SD	0.42±0.22	0.43±0.23	0.38±0.22	0.47
IL-4, Median (Q1,Q3)	0.44 (0.37, 0.54)	0.42 (0.36, 0.52)	0.5 (0.41, 0.62)	0.189
IL-8/CXCL8, Median (Q1,Q3)	0.05 (0.03, 0.09)	0.05 (0.03, 0.09)	0.02 (0.02, 0.16)	0.566
IP-10/CXCL10, Median (Q1,Q3)	0.15 (0.11, 0.22)	0.15 (0.11, 0.25)	0.13 (0.08, 0.16)	0.162
MCP-1/CCL2, Mean±SD	0.51±0.22	0.51±0.2	0.52±0.29	0.914

MCP-2/CCL8, Mean±SD	0.43±0.21	0.43±0.21	0.42±0.22	0.855
MCP-4/CCL13, Mean±SD	0.43±0.18	0.43±0.19	0.41±0.16	0.756
MDC/CCL22, Mean±SD	0.47±0.23	0.49±0.22	0.42±0.27	0.458
MIF, Mean±SD	0.5±0.18	0.51±0.17	0.47±0.23	0.621
MIG/CXCL9, Median (Q1,Q3)	0.16 (0.11, 0.26)	0.15 (0.09, 0.23)	0.16 (0.12, 0.31)	0.331
MIP-1alpha/CCL3, Median (Q1,Q3)	0.18 (0.12, 0.25)	0.17 (0.12, 0.23)	0.19 (0.13, 0.34)	0.55
MIP-3alpha/CCL20, Median (Q1,Q3)	0.07 (0.05, 0.11)	0.07 (0.05, 0.09)	0.08 (0.04, 0.11)	0.925
SCYB16/CXCL16, Median (Q1,Q3)	0.68 (0.54, 0.79)	0.68 (0.53, 0.78)	0.64 (0.58, 0.79)	0.963
SDF-1alpha+beta/CXCL12, Mean±SD	0.54±0.25	0.55±0.26	0.47±0.21	0.316
TARC/CCL17, Median (Q1,Q3)	0.3 (0.22, 0.4)	0.31 (0.22, 0.4)	0.3 (0.22, 0.43)	0.806
TECK/CCL25, Mean±SD	0.51±0.25	0.53±0.24	0.44±0.29	0.389
TNF-alpha, Median (Q1,Q3)	0.41 (0.29, 0.52)	0.38 (0.27, 0.52)	0.41 (0.38, 0.54)	0.622

Supplement Table 3. Tuberculosis Incidence among Participants According to different Biomarkers.

	Events (n)	Observation Time (Person-Years)	Rate per 100 Thousand Person-years	95% Confidence Interval
**All Participants**	11	30484	36.08	18.97-62.72
QuantiFERON-TB Gold-in Tube				
Negative	1	13,132	7.62	0.38-37.56
Positive	10	17352	57.63	29.27-102.70
Tuberculin skin test				
<10 mm	3	8911	33.67	8.56-91.63
⩾10 mm	8	21573	37.08	17.22-70.42
BasicFGF				
<14.37	5	22089	22.64	8.29-50.17
⩾14.37	6	8395	71.47	28.97-148.70
GM-CSF				
>1.69	1	9855	10.15	0.51-50.04
≤1.69	10	20629	48.48	24.62-86.41
IL-1ra				
<205.92	4	19214	20.82	6.62-50.22
⩾205.92	7	11270	62.11	27.17-12.29
I_309_CCL1				
>11.67	7	25983	26.94	11.78-53.29
≤11.67	4	4501	88.87	28.24-21.44
MPIF-1/CCL23				
>74.34	1	17583	5.69	0.28-28.05
≤74.34	10	12901	77.51	39.37-138.20
FGFbasic/GM-CSF				
<8.86	2	19381	10.32	1.73-34.09
⩾8.86	9	11103	81.06	39.53-148.80
FGFbasic/MPIF-1/CCL23				
<0.2516	3	25490	11.77	2.99-32.03
⩾0.2515	8	4994	160.19	74.40-304.20
Logistic model				
<0.2436	2	27330	7.32	12.27-241.80
⩾0.2436	9	3154	285.35	139.20-523.70
LASSO				
<0.2992	1	26516	3.77	0.19-18.60
⩾0.2992	10	3968	252.02	128.00-449.20

Supplement Table 4. Accuracy of Biomarkers in Predicting Risk of Progression to Tuberculosis Disease

Biomarkers	Cutoff	Sensitivity （95%CI）	Specificity （95%CI）	PPV （95%CI）	NPV （95%CI）	AUC （95%CI）
FGFbasic	≥14.37	0.545 (0.234, 0.833)	0.897 (0.579, 0.870)	0.375 (0.152, 0.646)	0.853 (0.689, 0.950)	0.713 (0.568, 0.832)
GM-CSF	≤1.69	0.909 (0.587, 0.998)	0.333 (0.191, 0.502)	0.278 (0.142, 0.452)	0.929 (0.661, 0.998)	0.607 (0.459, 0.742)
IL-1ra	≥205.92	0.636 (0.308, 0.891)	0.641 (0.472, 0.788)	0.333 (0.146, 0.570)	0.862 (0.683, 0.961)	0.652 (0.504, 0.781)
I_309_CCL1	≤11.67	0.364 (0.109, 0.692)	0.872 (0.726, 0.957)	0.444 (0.137, 0.788)	0.829 (0.679, 0.928)	0.628 (0.480, 0.761)
MPIF-1/CCL23	≤74.34	0.909 (0.587, 0.998)	0.615 (0.446, 0.766)	0.400 (0.211, 0.613)	0.960 (0.796, 0.999)	0.762 (0.621, 0.871)
FGFbasic/GM-CSF	≥8.86	0.818 (0.482, 0.977)	0.667 (0.498, 0.809)	0.409 (0.207, 0.636)	0.919 (0.781, 0.983)	0.710 (0.551, 0.859)
FGFbasic/MPIF-1/CCL23	≥0.2515	0.727 (0.390, 0.940)	0.872 (0.726, 0.957)	0.615 (0.316, 0.861)	0.919 (0.781, 0.983)	0.800 (0.628, 0.971)
Logistic model	≥0.2436	0.818 (0.482, 0.977)	0.923 (0.791, 0.984)	0.818 (0.482, 0.977)	0.949 (0.827, 0.994)	0.932 (0.854, 1.000)
LASSO model	≥0.2992	0.909 (0.587, 0.998)	0.949 (0.827, 0.994)	0.769 (0.462, 0.950)	0.973 (0.858, 0.999)	0.939 (0.856, 1.000)

Abbreviations: AUC, area under the curve; CI, confidence interval; NPV, negative predictive value; PPV, positive predictive value.

Supplement Table 5. Accuracy of Biomarkers in Predicting Risk of Progression to Tuberculosis Disease stratified by LASSO and Logistic Model.

LASSO Model					Logistic Model				
Threshold	Sensitivity	Specificity	PPV	NPV	Threshold	Sensitivity	Specificity	PPV	NPV
0.000	1.000	0.026	0.224	1.000	0.002	1.000	0.026	0.224	1.000
0.000	1.000	0.051	0.229	1.000	0.003	1.000	0.051	0.229	1.000
0.002	1.000	0.077	0.234	1.000	0.005	1.000	0.077	0.234	1.000
0.003	1.000	0.103	0.239	1.000	0.009	1.000	0.103	0.239	1.000
0.006	1.000	0.128	0.244	1.000	0.011	1.000	0.128	0.244	1.000
0.009	1.000	0.154	0.250	1.000	0.012	1.000	0.154	0.250	1.000
0.010	1.000	0.179	0.256	1.000	0.013	1.000	0.179	0.256	1.000
0.012	1.000	0.205	0.262	1.000	0.014	1.000	0.205	0.262	1.000
0.013	1.000	0.231	0.268	1.000	0.016	1.000	0.231	0.268	1.000
0.014	1.000	0.256	0.275	1.000	0.018	1.000	0.256	0.275	1.000
0.015	1.000	0.282	0.282	1.000	0.022	1.000	0.282	0.282	1.000
0.017	1.000	0.308	0.289	1.000	0.025	1.000	0.308	0.289	1.000
0.018	1.000	0.333	0.297	1.000	0.030	1.000	0.333	0.297	1.000
0.021	1.000	0.359	0.306	1.000	0.035	1.000	0.359	0.306	1.000
0.023	1.000	0.385	0.314	1.000	0.037	1.000	0.385	0.314	1.000
0.026	1.000	0.410	0.324	1.000	0.039	1.000	0.410	0.324	1.000
0.029	1.000	0.436	0.333	1.000	0.039	1.000	0.436	0.333	1.000
0.030	1.000	0.462	0.344	1.000	0.041	1.000	0.462	0.344	1.000
0.031	1.000	0.487	0.355	1.000	0.045	1.000	0.487	0.355	1.000
0.034	1.000	0.513	0.367	1.000	0.048	1.000	0.513	0.367	1.000
0.039	1.000	0.538	0.379	1.000	0.053	1.000	0.538	0.379	1.000
0.042	1.000	0.564	0.393	1.000	0.065	1.000	0.564	0.393	1.000
0.047	0.909	0.564	0.370	0.957	0.073	1.000	0.590	0.407	1.000
0.053	0.909	0.590	0.385	0.958	0.078	1.000	0.615	0.423	1.000
0.057	0.909	0.615	0.400	0.960	0.086	0.909	0.615	0.400	0.960
0.065	0.909	0.641	0.417	0.962	0.094	0.909	0.641	0.417	0.962
0.069	0.909	0.667	0.435	0.963	0.106	0.909	0.667	0.435	0.963
0.073	0.909	0.692	0.455	0.964	0.124	0.909	0.692	0.455	0.964
0.081	0.909	0.718	0.476	0.966	0.133	0.909	0.718	0.476	0.966
0.089	0.909	0.744	0.500	0.967	0.145	0.909	0.744	0.500	0.967
0.097	0.909	0.769	0.526	0.968	0.160	0.909	0.769	0.526	0.968
0.107	0.909	0.795	0.556	0.969	0.166	0.909	0.795	0.556	0.969
0.114	0.909	0.821	0.588	0.970	0.187	0.909	0.821	0.588	0.970
0.117	0.909	0.846	0.625	0.971	0.207	0.909	0.846	0.625	0.971
0.167	0.909	0.872	0.667	0.971	0.210	0.818	0.846	0.600	0.943
0.229	0.909	0.897	0.714	0.972	0.222	0.818	0.872	0.643	0.944
0.250	0.909	0.923	0.769	0.973	0.232	0.818	0.897	0.692	0.946

0.299	0.909	0.949	0.833	0.974	0.235	0.818	0.923	0.750	0.947
0.340	0.818	0.949	0.818	0.949	0.244	0.818	0.949	0.818	0.949
0.450	0.727	0.949	0.800	0.925	0.269	0.727	0.949	0.800	0.925
0.569	0.636	0.949	0.778	0.902	0.382	0.636	0.949	0.778	0.902
0.593	0.545	0.949	0.750	0.881	0.551	0.545	0.949	0.750	0.881
0.724	0.545	0.974	0.857	0.884	0.670	0.455	0.949	0.714	0.860
0.859	0.455	0.974	0.833	0.864	0.748	0.455	0.974	0.833	0.864
0.886	0.455	1.000	1.000	0.867	0.811	0.455	1.000	1.000	0.867
0.898	0.364	1.000	1.000	0.848	0.881	0.364	1.000	1.000	0.848
0.929	0.273	1.000	1.000	0.830	0.943	0.273	1.000	1.000	0.830
0.967	0.182	1.000	1.000	0.813	0.972	0.182	1.000	1.000	0.813
0.981	0.091	1.000	1.000	0.796	0.989	0.091	1.000	1.000	0.796

Abbreviations: NPV, negative predictive value; PPV, positive predictive value.
