# Supplementary figures and images for: Predicting tuberculosis progression in school contacts: novel host biomarkers for early risk assessment
Source: Front Cell Infect Microbiol. 2025 Sep 1;15:1635486. doi: 10.3389/fcimb.2025.1635486 (PMC12434022; doi:10.3389/fcimb.2025.1635486)

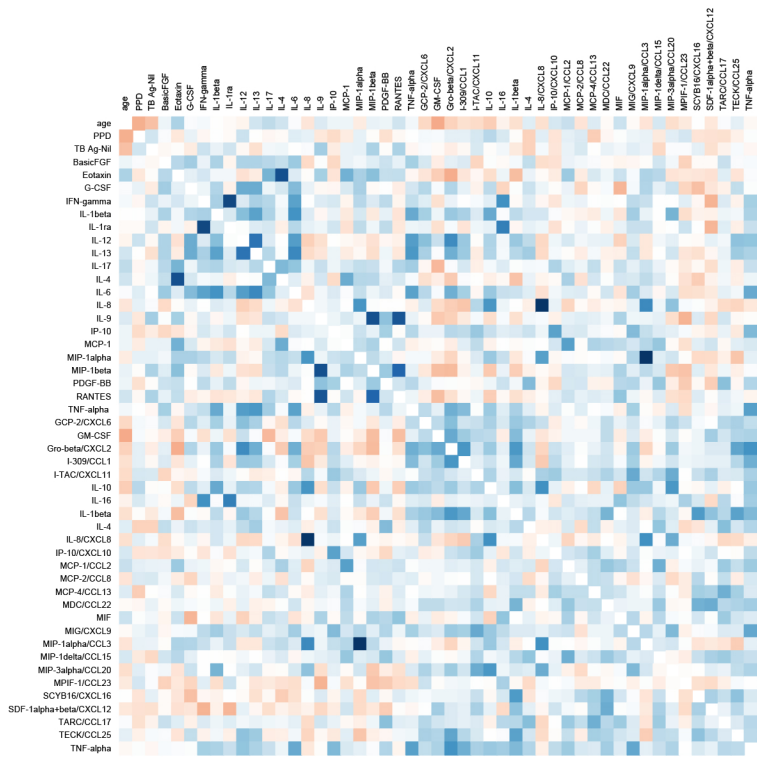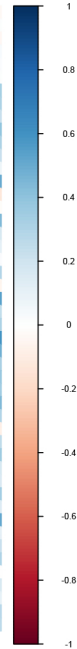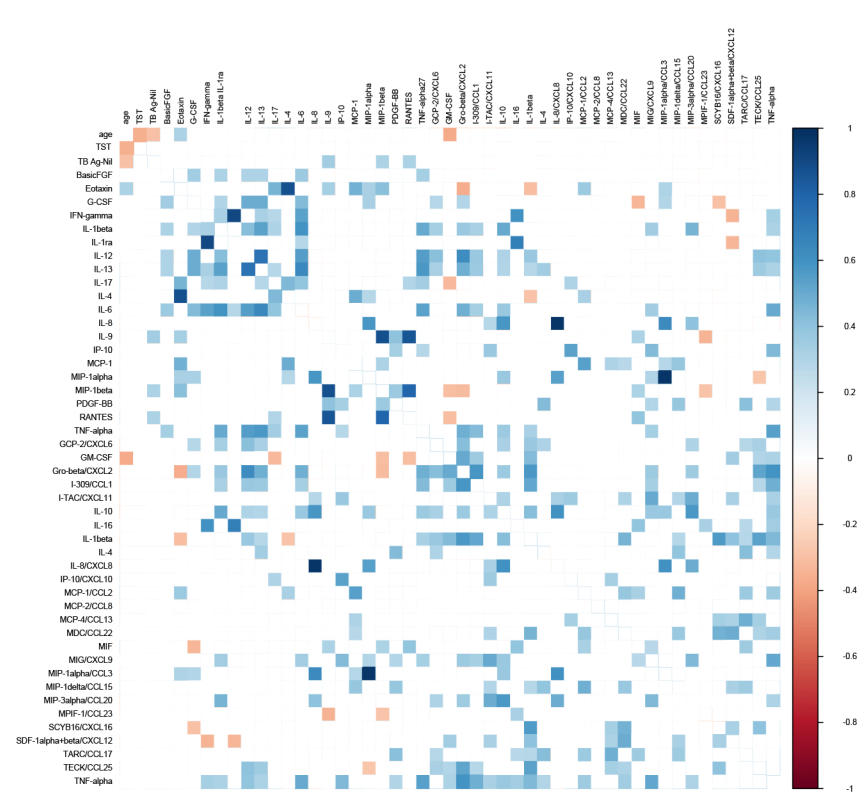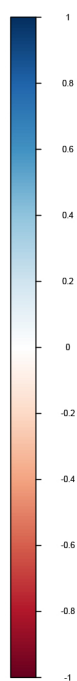

Supplement: Supplementary Figure 2 — CPCA plot of significantly different biomarkers between tuberculosis and non-progressors: PCA shows that FGFbasic, GM-CSF, FGFbasic, IL-1ra, I-309/CCL1, MPIF-1/CCL23 can clearly distinguish between tuberculosis and non-tuberculosis with no overlap. [file DataSheet2.pdf]

PCA - Tuberculosis vs Non-tuberculosis

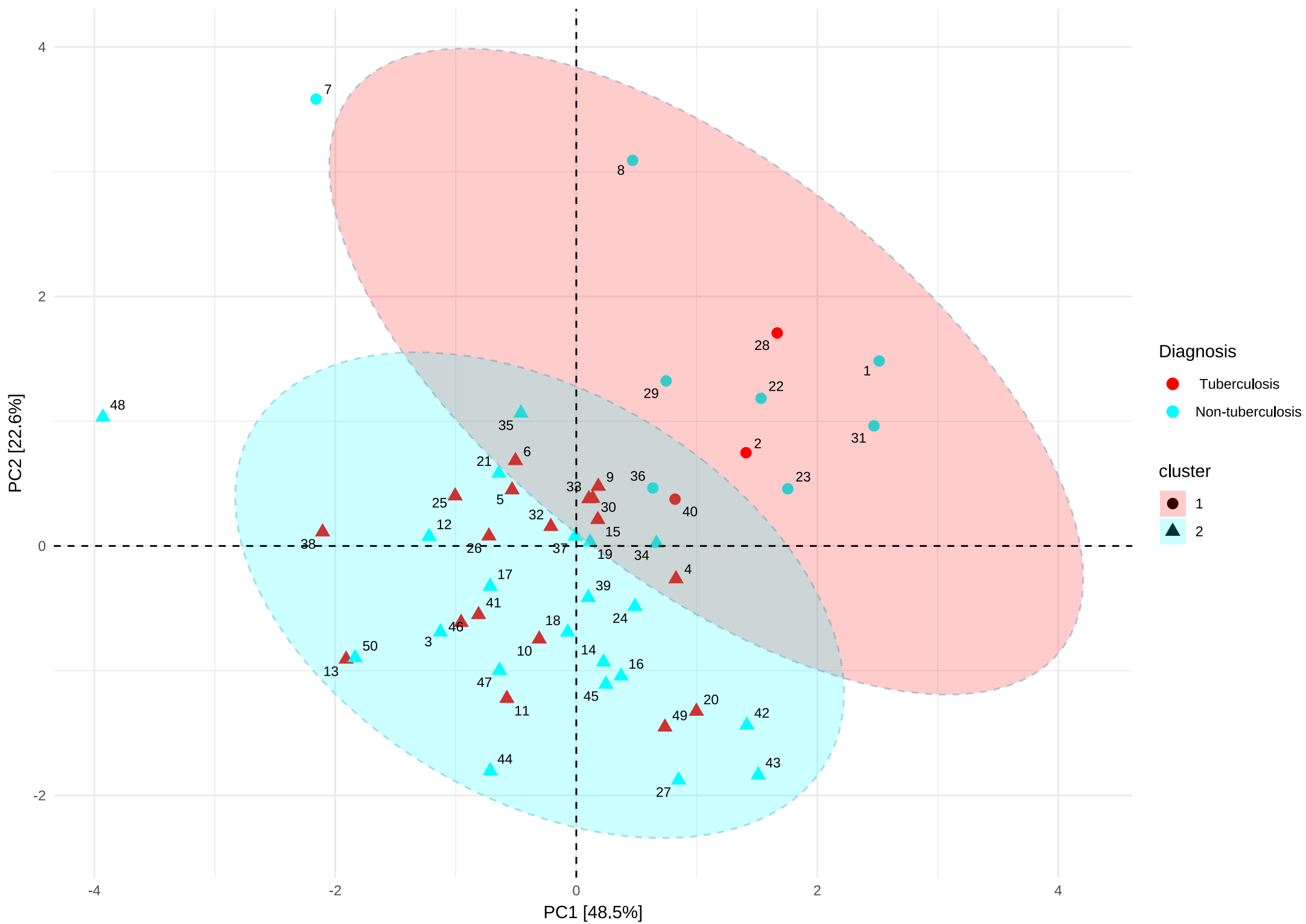

Supplement: Supplementary file 3 [file DataSheet3.pdf]
